# Supplementary material for: Extracts and Residues of Common Ragweed (Ambrosia artemisiifolia L.) Cause Alterations in Root and Shoot Growth of Crops
Source: Plants (Basel). 2023 Apr 26;12(9):1768. doi: 10.3390/plants12091768 (PMC10180818; doi:10.3390/plants12091768)
Supplement: Supplementary file 1 [file plants-12-01768-s001.zip › plants-2331697-supplementary.pdf]

Table S1: 20 Most abundant chemical constituents of the essential oil of ragweed

Compound	Classification	Percentage of total
Germacrene D	sesquiterpene	48.9 %
Germacrene B	sesquiterpene	7.8 %
β-Caryophyllene	sesquiterpene	3.8 %
β-Myrcene	terpene	3.7 %
Ocimene	monoterpene	3.7 %
Phytol	diterpene	3.3 %
D-Limonene	monoterpene	2.5 %
Borneol	monoterpene	2.0 %
cis-Lanceol	sesquiterpene	1.7 %
Naphtalene (γ-Muurolene)	terpene	1.6 %
α-Caryophyllene	sesquiterpene	1.6 %
α-Bergamotene	sesquiterpene	1.6 %
β-Elemene	monoterpene	1.3 %
β-Panasinsene	sesquiterpene	1.1 %
1S-α-Pinene	monoterpene	1.1 %
Guaiol	sesquiterpene	1.0 %
β-Pinene	monoterpene	0.9 %
Δ-Elemene	monoterpene	0.9 %
Δ-Cadinene	sesquiterpene	0.9 %
β-Selinene	sesquiterpene	0.8 %

Table S2: Chemical constituents of the aqueous extracts (shoot and root) of f ragweed

Substance	Substance type
(4-Aminobutyl)guanidine	amino compound
Ambrosic acid	oxane
6,15-Dihydroxy-1(10),4,11(13)-germacratrien-12,8-olide	germacranolide
Artemisinin	sesquiterpene lactone
1,4-Dihydroxy-11(13)-pseudoguaien-12,6-olide	sesquiterpene lactone
1,8-Dihydroxy-5-oxo-4(15),10(14),11(13)-germacratrien-12,6-olide	germacranolide
Psilostachyin	sesquiterpene lactone
Isabelin	sesquiterpene lactone
4-Oxo-3,4-secoambrosan-12,6-olid-3-oic acid	sesquiterpene lactone
5,6-Dihydroxy-4(15),11(13)-germacradien-12,8-olid-14-oic acid	germacranolide
6,15-Dihydroxy-4,11(13)-germacradien-12,8-olid-14-oic acid	germacranolide
3,4-Dihydroxy-11(13)-pseudoguaien-12,6-olide	γ-lactone
4(15)-Eudesmene-1,6-diol	sesquiterpenoid
5,8,9,10-Daucanetetrol	hydroxy steroid
9-Hydroxy-4-guaien-12,6-olide	sesquiterpene lactone
4,9-Dihydroxy-10(14),11(13)-guaiadien-12-oic acid	sesquiterpenoid
4(15)-Eudesmene-1,6-diol;	sesquiterpenoid

Table S3: Germination rate (%) and root length (cm) of soybean, wheat, maize, and ragweed (mean ± standard deviation) seven days after seeding; germination rate: n = 100; root length: n = 25; significance levels: n.s. = not significant, * $p < 0.05$, ** $p < 0.01$, *** $p < 0.001$ refer to the untreated control

		**Shoot extract (ST)**			**Root extract (RT)**			**Essential Oil (EO)**		**Residues (Resi)**		
	**Control**	**1%**	**10%**	**100%**	**1%**	**10%**	**100%**	**0.5 %**	**1%**	**1%**	**5%**	**10%**
**Soybean**												
Germination rate (%)	**94**	87	88	75	88	82	87	77	82	98	94	94
Root length (cm)	**5.7 ± 1.3**	2.7 ± 0.9	2.6 ± 0.9	2.5 ± 0.8	4.3 ± 1.1	2.3 ± 0.7	2.8 ± 1.4	4.7 ± 1.1	4.5 ± 0.9	6.0 ± 1.9	5.6 ± 1.6	5.2 ± 1.3
		***	***	***	**	***	***	**	***	n.s.	n.s.	n.s.
**Wheat**												
Germination rate (%)	**100**	99	92	93	89	97	97	96	99	98	97	98
Root length (cm)	**10.3 ± 0.8**	9.7 ± 0.9	8.5 ± 1.8	4.1 ± 1.4	7.4 ± 1.2	6.2 ± 1.4	5.4 ± 1.3	5.1 ± 1.3	2.6 ± 0.9	10.7 ± 1.4	8.6 ± 1.0	6.1 ± 1.3
		n.s.	***	***	***	***	***	***	***	n.s.	*	***
**Maize**												
Germination rate (%)	**98**	84	88	83	82	88	92	84	86	92	91	92
Root length (cm)	**11.4 ± 1.9**	10.9 ± 2.1	7.6 ± 2.1	6.7 ± 3.3	9.8 ± 3.2	10.9 ± 4.1	8.7 ± 2.7	4.3 ± 1.8	8.1 ± 2.5	9.8 ± 2.6	10.5 ± 2.3	10.2 ± 2.8
		n.s.	***	***	n.s.	n.s.	n.s.	***	***	n.s.	n.s.	n.s.
**Ragweed**												
Germination rate (%)	**80**	72	78	74	49	46	47	74	75	73	76	55
Root length (cm)	**9.7 ± 5.0**	12.4 ± 3.2	15.9 ± 9.6	10.0 ± 6.4	6.8 ± 1.9	6.2 ± 2.3	6.1 ± 2.1	5.5 ± 3.8	6.3 ± 4.0	4.9 ± 1.5	4.8 ± 0.7	3.3 ± 1.1
		n.s.	n.s.	n.s.	n.s.	n.s.	n.s.	**	n.s.	*	**	***

Table S4: Aboveground dry matter (mg) and root mass (mg) of soybean, wheat, maize, and ragweed in dependency of the treatment (ST, RT, EO, and Resi) and the concentration of the treatment 7, 10, and 24 days after seeding (DAS), (mean ± standard deviation); n = 10; significance levels: n.s. = not significant, * $p < 0.05$, ** $p < 0.01$, *** $p < 0.001$ refer to the untreated control

Aboveground dry matter (mg)

		Shoot extract (ST)						Root extract (RT)						Essential Oil (EO)				Residues (Resi)					
	**Control**	**1 %**		**10 %**		**100 %**		**1 %**		**10 %**		**100 %**		**0.5 %**		**1 %**		**1 %**		**5 %**		**10 %**	
**Soybean**																							
10 DAS	**139 ± 19**	195 ± 28	***	167 ± 27	n.s.	172 ± 44	n.s.	195 ± 22	***	195 ± 26	***	174 ± 22	n.s.	181 ± 20	**	142 ± 37	n.s.	137 ± 48	n.s.	149 ± 59	n.s.	136 ± 121	n.s.
17 DAS	**161 ± 33**	179 ± 14	n.s.	165 ± 13	n.s.	182 ± 23	n.s.	149 ± 41	n.s.	175 ± 35	n.s.	171 ± 42	n.s.	144 ± 31	n.s.	121 ± 43	n.s.	159 ± 27	n.s.	163 ± 34	n.s.	139 ± 74	n.s.
24 DAS	**241 ± 32**	202 ± 24	n.s.	129 ± 68	***	121 ± 66	***	168 ± 90	n.s.	85 ± 68	***	110 ± 63	***	131 ± 82	**	87 ± 78	***	118 ± 64	***	128 ± 71	**	81 ± 74	***
**Wheat**																							
10 DAS	**20 ± 13**	30 ± 7 a	n.s.	18 ± 5 a	n.s.	23 ± 8 b	n.s.	22 ± 8	n.s.	21 ± 7	n.s.	24 ± 8	n.s.	11 ± 9	n.s.	8 ± 10	*	24 ± 6	n.s.	14 ± 12	n.s.	15 ± 7	n.s.
17 DAS	**29 ± 4**	28 ± 6	n.s.	35 ± 6	n.s.	51 ± 2	***	35 ± 18	n.s.	32 ± 7	n.s.	30 ± 14	n.s.	27 ± 9	n.s.	16 ± 8	***	29 ± 7	n.s.	27 ± 7	n.s.	25 ± 19	n.s.
24 DAS	**50 ± 8**	44 ± 7	n.s.	46 ± 21	n.s.	54 ± 7	n.s.	43 ± 18	n.s.	48 ± 14	n.s.	55 ± 11	n.s.	34 ± 19	n.s.	12 ± 11	***	47 ± 15	n.s.	48 ± 26	n.s.	34 ± 19	**
**Maize**																							
10 DAS	**71 ± 11**	61 ± 26	n.s.	71 ± 13	n.s.	80 ± 16	n.s.	61 ± 7	n.s.	68 ± 25	n.s.	48 ± 19	**	60 ± 19	n.s.	60 ± 15	n.s.	60 ± 12	n.s.	53 ± 7	n.s.	33 ± 8	***
17 DAS	**116 ± 20**	142 ± 25	n.s.	158 ± 46	*	146 ± 52	n.s.	133 ± 18	n.s.	154 ± 23	*	137 ± 45	n.s.	171 ± 52	**	129 ± 50	n.s.	119 ± 18	n.s.	107 ± 43	n.s.	80 ± 35	*
24 DAS	**244 ± 50**	223 ± 40	n.s.	166 ± 95	n.s.	156 ± 89	*	157 ± 16	***	187 ± 22	n.s.	132 ± 73	***	150 ± 51	***	187 ± 32	**	157 ± 48	***	94 ± 15	***	32 ± 46	***
**Ragweed**																							
10 DAS	**8 ± 1**	8 ± 4	n.s.	12 ± 9	n.s.	11 ± 4	n.s.	12 ± 7	n.s.	11 ± 5	n.s.	9 ± 2	n.s.	11 ± 6	n.s.	10 ± 4	n.s.	9 ± 5	n.s.	8 ± 12	n.s.	0 ± 0	***
17 DAS	**16 ± 5**	12 ± 4	n.s.	28 ± 10	**	32 ± 10	***	21 ± 14	n.s.	23 ± 8	n.s.	7 ± 5	n.s.	22 ± 12	n.s.	35 ± 16	**	1 ± 1	***	1 ± 2	**	0 ± 0	***
24 DAS	**19 ± 2**	7 ± 3	n.s.	53 ± 20	***	58 ± 18	***	20 ± 11	n.s.	14 ± 12	n.s.	7 ± 6	***	6 ± 4	***	12 ± 9	n.s.	7 ± 4	n.s.	2 ± 2	***	2 ± 2	***

Root mass (mg)

		Shoot extract (ST)						Root extract (RT)						Essential Oil (EO)				Residues (Resi)					
	**Control**	**1 %**		**10 %**		**100 %**		**1 %**		**10 %**		**100 %**		**0.5 %**		**1 %**		**1 %**		**5 %**		**10 %**	
**Soybean**																							
10 DAS	**15 ± 3**	13 ± 9	n.s.	13 ± 8	n.s.	10 ± 6	n.s.	12 ± 5	n.s.	7 ± 4	n.s.	15 ± 5	n.s.	17 ± 3	n.s.	11 ± 6	n.s.	14 ± 4	n.s.	4 ± 3	***	3 ± 2	***
17 DAS	**30 ± 4**	37 ± 10	n.s.	32 ± 7	n.s.	19 ± 7	*	29 ± 9	n.s.	31 ± 14	n.s.	25 ± 10	n.s.	17 ± 10	**	28 ± 8	n.s.	24 ± 8	***	9 ± 6	***	6 ± 4	***
24 DAS	**68 ± 7**	44 ± 16	n.s.	24 ± 23	***	24 ± 16	***	48 ± 26	n.s.	32 ± 20	***	14 ± 18	***	38 ± 24	*	24 ± 30	***	15 ± 15	**	6 ± 5	***	9 ± 9	***
**Wheat**																							
10 DAS	**10 ± 1**	13 ± 2	n.s.	12 ± 3	n.s.	11 ± 3	n.s.	13 ± 2	*	11 ± 3	n.s.	12 ± 3	n.s.	10 ± 6	n.s.	5 ± 4	*	12 ± 2	n.s.	7 ± 5	n.s.	7 ± 3	n.s.
17 DAS	**23 ± 4**	20 ± 3	n.s.	20 ± 4	n.s.	24 ± 4	n.s.	19 ± 5	n.s.	24 ± 5	n.s.	23 ± 7	n.s.	18 ± 6	n.s.	11 ± 2	***	24 ± 8	n.s.	15 ± 2	n.s.	8 ± 5	***
24 DAS	**38 ± 3**	38 ± 4	n.s.	29 ± 9	*	24 ± 7	***	33 ± 6	n.s.	30 ± 3	**	27 ± 8	***	25 ± 14	*	9 ± 10	***	35 ± 10	n.s.	18 ± 10	***	16 ± 10	***
**Maize**																							
10 DAS	**133 ± 27**	117 ± 12	n.s.	154 ± 35	n.s.	159 ± 47	n.s.	123 ± 25	n.s.	143 ± 17	n.s.	125 ± 28	n.s.	161 ± 63	n.s.	145 ± 28	n.s.	129 ± 20	n.s.	136 ± 7	n.s.	142 ± 40	n.s.
17 DAS	**141 ± 38**	120 ± 40	n.s.	154 ± 23	n.s.	170 ± 33	n.s.	137 ± 27	n.s.	146 ± 36	n.s.	160 ± 32	n.s.	188 ± 41	**	154 ± 24	n.s.	162 ± 46	n.s.	167 ± 23	n.s.	159 ± 29	n.s.
24 DAS	**153 ± 19**	176 ± 48	n.s.	228 ± 53	**	151 ± 81	n.s.	128 ± 36	n.s.	110 ± 63	n.s.	142 ± 84	n.s.	182 ± 36	n.s.	152 ± 36	n.s.	166 ± 62	n.s.	110 ± 18	*	101 ± 63	**
**Ragweed**																							
10 DAS	**0.6 ± 0.3**	2 ± 1	n.s.	3 ± 2	n.s.	3 ± 3	n.s.	3 ± 2	*	2 ± 2	n.s.	1 ± 1	n.s.	3 ± 1	n.s.	3 ± 2	n.s.	2 ± 1	n.s.	0.3 ± 0.4	n.s.	0 ± 0	**
17 DAS	**8 ± 6**	9 ± 4	n.s.	13 ± 7	n.s.	12 ± 4	n.s.	10 ± 6	n.s.	9 ± 3	n.s.	2 ± 2	**	2 ± 1	n.s.	4 ± 4	n.s.	0.2 ± 0.3	***	0.3 ± 0.7	***	0 ± 0	***
24 DAS	**12 ± 5**	10 ± 3	n.s.	29 ± 6	***	35 ± 14	***	11 ± 4	n.s.	8 ± 7	n.s.	3 ± 2	***	8 ± 4	n.s.	15 ± 9	n.s.	2 ± 1	**	1 ± 1	***	0.2 ± 0.3	***

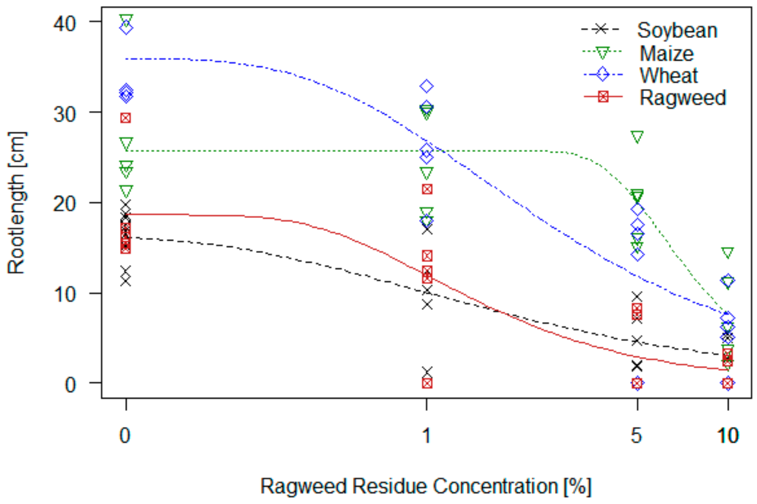

Figure S1. Reduction in root length (cm) of soybean, wheat, maize and ragweed after a 24-days exposure to ragweed residues; logistic regression on basis of most parsimonious model based on Weibull probability distribution, n = 10.
